# Time-Trend Analysis of Low Birthweight in Greece: Mapping a Heavy Public Health Burden

**DOI:** 10.7759/cureus.81657

**Published:** 2025-04-03

**Authors:** Nikolaos Vlachadis, Dimos Sioutis, Chryssi Christodoulaki, Nikolaos Machairiotis, Dimitrios Panagiotopoulos, Konstantinos Louis, Maria Siori, Amalia Koutsoukou, Anastasia Barbouni, Periklis Panagopoulos

**Affiliations:** 1 Department of Obstetrics and Gynecology, General Hospital of Messinia, Kalamata, GRC; 2 Third Department of Obstetrics and Gynecology, National and Kapodistrian University of Athens, Medical School, Attiko Hospital, Athens, GRC; 3 Department of Obstetrics and Gynecology, General Hospital of Messinia, Kyparissia, GRC; 4 Vyronas Health Center, National Health System, Athens, GRC; 5 Laboratory of Hygiene and Epidemiology, Department of Public and Community Health, University of West Attica, Athens, GRC

**Keywords:** births, birthweight, greece, low birthweight, time trends, very low birth weight

## Abstract

Introduction: Low birthweight is a critical determinant of neonatal and infant mortality and is further associated with several long-term adverse health outcomes. This study aims to comprehensively analyze low birthweight rate (LBWR) trends in Greece from 1980 to 2023.

Materials and methods: National official data on live births in Greece from 1980 to 2023 were obtained from the Hellenic Statistical Authority, based on birth certificate records. A total of 4,593,229 live births were analyzed and categorized by birthweight. The annual LBWR (birthweight < 2,500 g) and very low birthweight rate (VLBWR) (birthweight < 1,500 g) were calculated per 100 total live births. Additionally, the annual moderately low birthweight rate (MLBWR) was computed separately for two subgroups: 1,500-1,999 g and 2,000-2,499 g. Time trends for these rates were evaluated using joinpoint regression analysis, and the annual percent change (APC) was calculated with 95% confidence intervals (95% CI).

Results: After a decade of stability from 1980 to 1990, the LBWR in Greece entered a 20-year period of consistent increase (1990-2010), with an APC of 2.3 (95% CI: 1.9 to 4.9). In the most recent period (2010-2023), the LBWR has plateaued at high levels. The LBWR reached its lowest point in 1982 at 5.58% and increased by 80%, reaching historically high levels in 2022 and 2023 at 10.07% and 10.02%, respectively. Since 2008, it has consistently remained above 9%. The VLBWR exhibited a steady upward trend throughout the entire 1980-2023 period with an APC of 0.9 (95% CI: 0.7 to 1.1) and increased from a low of 0.70% in 1980 to a peak of 1.20% in 2010. For the 2,000-2,499 g category, the MLBWR rose steadily over three decades (1980-2010: APC = 2.0, 95% CI: 1.8 to 2.3) and remained essentially unchanged from 2010 to 2023. In contrast, the MLBWR for the 1,500-1,999 g group showed a non-significant trend during 1980-1990, followed by a rapid increase between 1990 and 2001 (APC = 3.9, 95% CI: 2.9 to 7.9), which continued at a slower pace from 2001 to 2023 (APC = 1.0, 95% CI: 0.5 to 1.3).

Conclusions: During 1980-2023, the LBWR increased by 80%, with significant rising trends in the 1990s and 2000s, resulting in Greece having the highest rates among high-income countries. Whereas the overall LBWR has stabilized since 2010, there has been a continued rise in the proportion of neonates with birthweight < 2,000 g, who face the highest risk of adverse outcomes. Continued monitoring of LBWR is essential, alongside investments in the implementation of effective, targeted interventions.

## Introduction

Low birthweight (LBW) is defined as the birthweight of live newborns < 2,500 g, regardless of gestational age, and is a critical determinant of neonatal and infant mortality and morbidity. LBW includes preterm neonates (born < 37 weeks of gestation), those with fetal growth restriction (FGR) (< 10th percentile of weight for gestational age), or a combination of both. LBW is a vital public health measure recognized by the World Health Organization (WHO) as a key indicator for assessing national populations and facilitating cross-country comparisons, particularly in settings where accurate gestational age assessment is challenging [1-3].

LBW is a significant public health issue globally, linked to both immediate and long-term consequences that impact human capital. Over 80% of neonatal deaths occur in LBW newborns, with approximately two-thirds of these being preterm. LBW infants face higher risks of mortality and long-term adverse outcomes, including neurodevelopmental impairments and an increased likelihood of adult chronic diseases, such as diabetes and cardiovascular disease. Addressing LBW is essential for improving health outcomes and reducing the burden of disease across the lifespan [1,4-6].

Factors influencing LBW include extremes of maternal age (adolescent pregnancy and advanced maternal age), multiple pregnancies, obstetric complications, chronic maternal conditions, infections, and poor maternal nutritional status. Additional contributors include exposure to environmental factors, as well as tobacco and substance use. LBW is also closely linked to socioeconomic conditions, with a documented higher prevalence among socially deprived families and areas, independent of other physiological or disease-related factors [1,4-6].

Globally, it is estimated that the number of LBW newborns decreased from approximately 22 million to 20 million between 2000 and 2020, representing a decline from about 16.6% to 14.7%. During this period, the proportion of LBW live births in developed countries saw minimal improvement, dropping only slightly from 7.3% to 7.2%. In contrast, more significant reductions were observed in regions with the highest burden of LBW, such as South Asia and sub-Saharan Africa [1].

The WHO has endorsed a comprehensive implementation plan aimed at achieving a 30% reduction in LBW live births between 2012 and 2025, positioning LBW as a key indicator of progress toward global health targets. The significance of LBW as a major health indicator lies in its ability to reflect not only infant health but also maternal risk factors while projecting future health implications for the adult population [4-6]. This has motivated us to study the time trends of LBW rates in the Greek population since 1980. This research is particularly important given the reported high levels of prematurity in the country, as preterm birth is the primary factor contributing to LBW [7]. Close monitoring of these trends is essential for identifying population health needs and can inform the development of effective public health policies.

## Materials and methods

The study was conducted at the Third Department of Obstetrics and Gynecology, National and Kapodistrian University of Athens, Medical School, located in Attiko Hospital, Athens, Greece.

Study population

The study encompassed all live births recorded in Greece from 1980 to 2023, stratified by birthweight. Data were sourced from the Hellenic Statistical Authority [8], utilizing birth certificate records.

Inclusion and exclusion criteria

During the study period (1980-2023), a total of 4,605,769 live births were documented in Greece. Of these, 4,593,229 live births (99.73%) with complete birthweight data were included in the analysis. The remaining 12,540 births (0.27%) were excluded due to missing or unregistered birthweight information.

Study parameters

The study parameters focused on evaluating birthweight rates as key indicators. The low birthweight rate (LBWR) was calculated as the number of live births weighing less than 2,500 g per 100 total live births. Additionally, the very low birthweight rate (VLBWR) was determined, which is defined as the number of live births weighing less than 1,500 g per 100 total live births. Furthermore, the moderately low birthweight rate (MLBWR) was computed separately for two categories: the proportion of newborns weighing 1,500-1,999 g and those weighing 2,000-2,499 g, each per 100 total live births. These rates were calculated annually over the study period from 1980 to 2023.

Statistical analysis

Data analysis was performed using Microsoft Excel 2010 (Microsoft Corporation, Redmond, Washington, United States). Trend analysis was conducted using the Joinpoint Regression Program, version 5.2.0 (National Cancer Institute, United States). This software identifies joinpoints, which define specific time segments where statistically significant changes in trends occur. The analysis computed the annual percent change (APC) for each segment between two joinpoints, allowing for a maximum of seven segments. Results were reported with 95% confidence intervals (95% CI), and statistical significance was determined at a p-value threshold of < 0.05.

## Results

**Figure 1:**
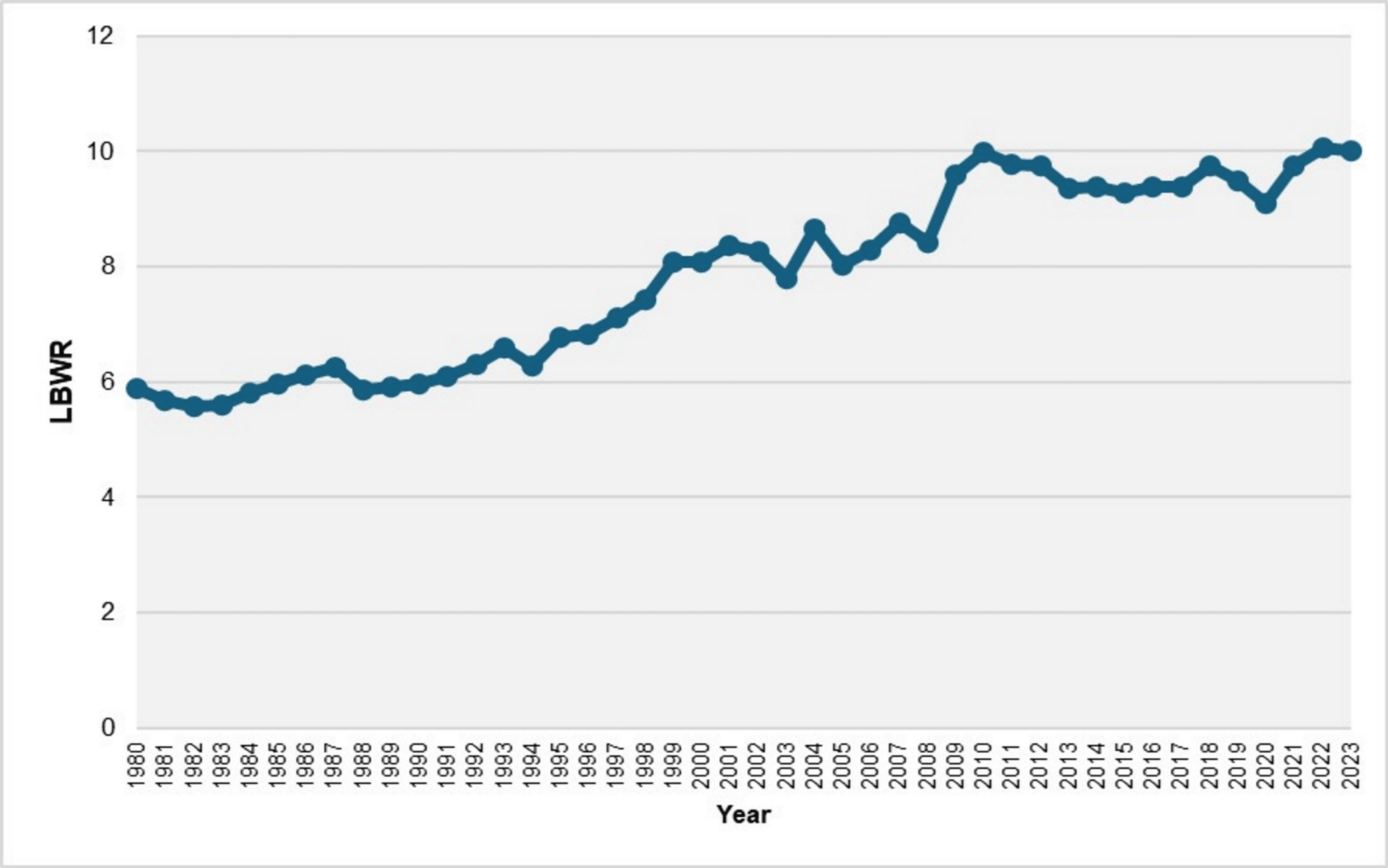
Low birthweight rate (LBWR) (per 100 live births) in Greece, 1980-2023 Low birthweight is defined as a birthweight < 2,500 g.

**Figure 2:**
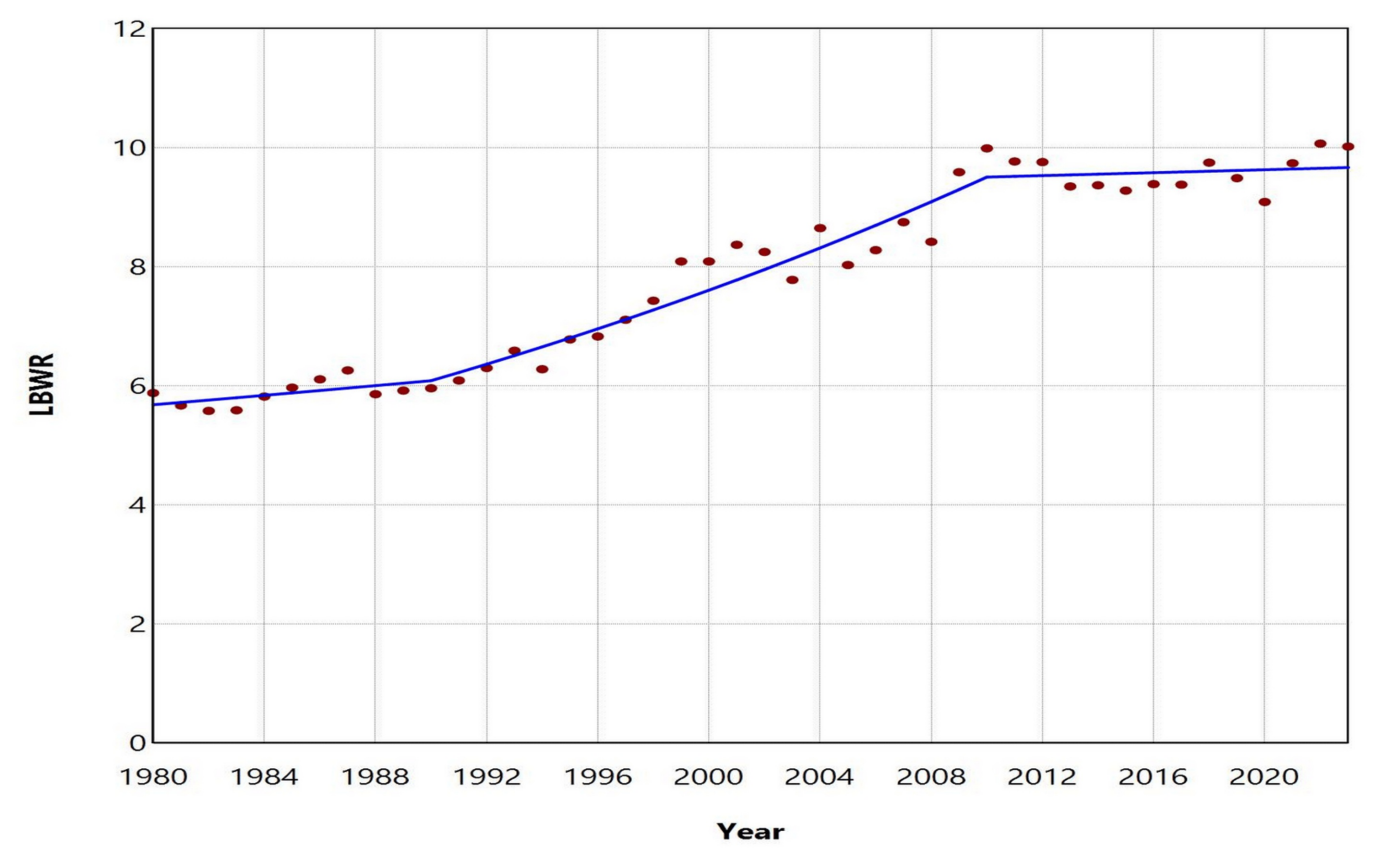
Trends in low birthweight rate (LBWR) in Greece, 1980-2023 Solid lines represent the estimated trends derived from joinpoint regression analysis.

During the period 1980-2023, among 4,593,229 live births with complete birthweight data, there were 353,090 LBW neonates (< 2,500 grams), resulting in an overall LBWR of 7.69%. After decreasing as low as 5.58% in 1982, the LBWR increased by 80% to reach record highs of 10.07% and 10.02% in 2022 and 2023, respectively. Since 2008, the LBWR has consistently remained above 9%. Analysis of temporal trends revealed three distinct periods. The first period, 1980-1990, showed no significant change in LBWR (annual percent change (APC) = 0.7, 95% CI: -2.6 to 1.7, p = 0.364). This was followed by a 20-year period of consistent increase (1990-2010), with an APC of 2.3 (95% CI: 1.9 to 4.9, p = 0.029). In the most recent period, 2010-2023, the LBWR stabilized at high levels (APC = 0.1, 95% CI: -0.8 to 0.9, p = 0.721) (Figures 1, 2; Table 1).

**Table 1:** Trends in low birthweight rate in Greece, 1980-2023 Trends were calculated using joinpoint regression analysis.

Segment	Annual percent change	95% confidence interval	P-value
1980-1990	0.7	-2.6 to 1.7	0.364
1990-2010	2.3	1.9 to 4.9	0.029
2010-2023	0.1	-0.8 to 0.9	0.721

**Figure 3:**
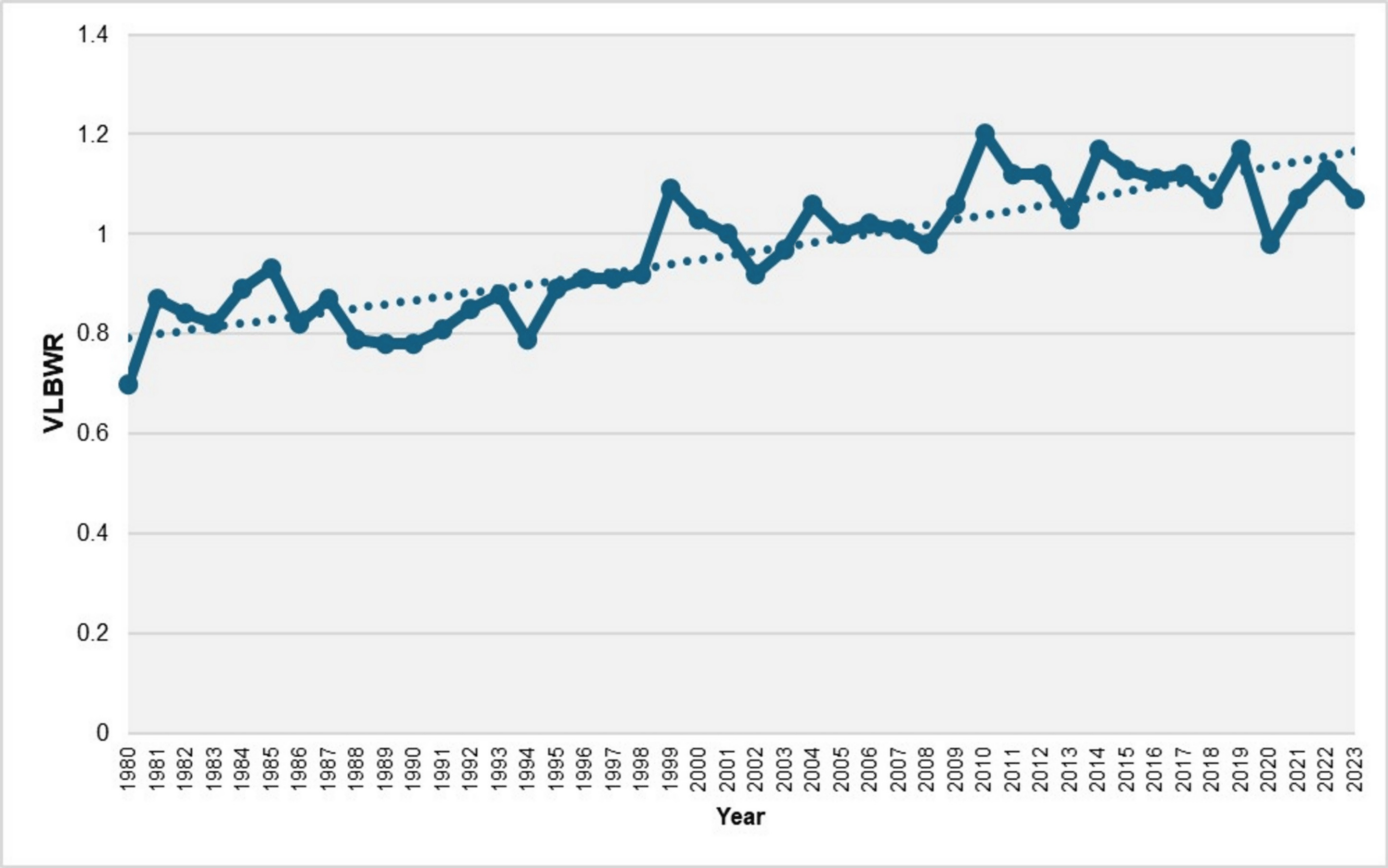
Trend in very low birthweight rate (VLBWR) (per 100 live births) in Greece, 1980-2023 Very low birthweight is defined as a birthweight < 1,500 g.

From 1980 to 2023, the total number of very LBW newborns (< 1,500 grams) was 44,090, resulting in an overall VLBWR of 0.96%. The VLBWR increased by approximately 70%, rising from a low of 0.70% in 1980 to a peak of 1.20% in 2010. In 2022 and 2023, the rates were 1.13% and 1.07%, respectively. Since 2008, the VLBWR has consistently remained above 1%, except for 2020, which saw a rate of 0.98%, a 16% decline from 1.17% in 2019. Throughout the entire study period (1980-2023), the VLBWR exhibited a steady upward trend, with an APC of 0.9 (95% CI: 0.7 to 1.1, p < 0.001) (Figure 3).

**Figure 4:**
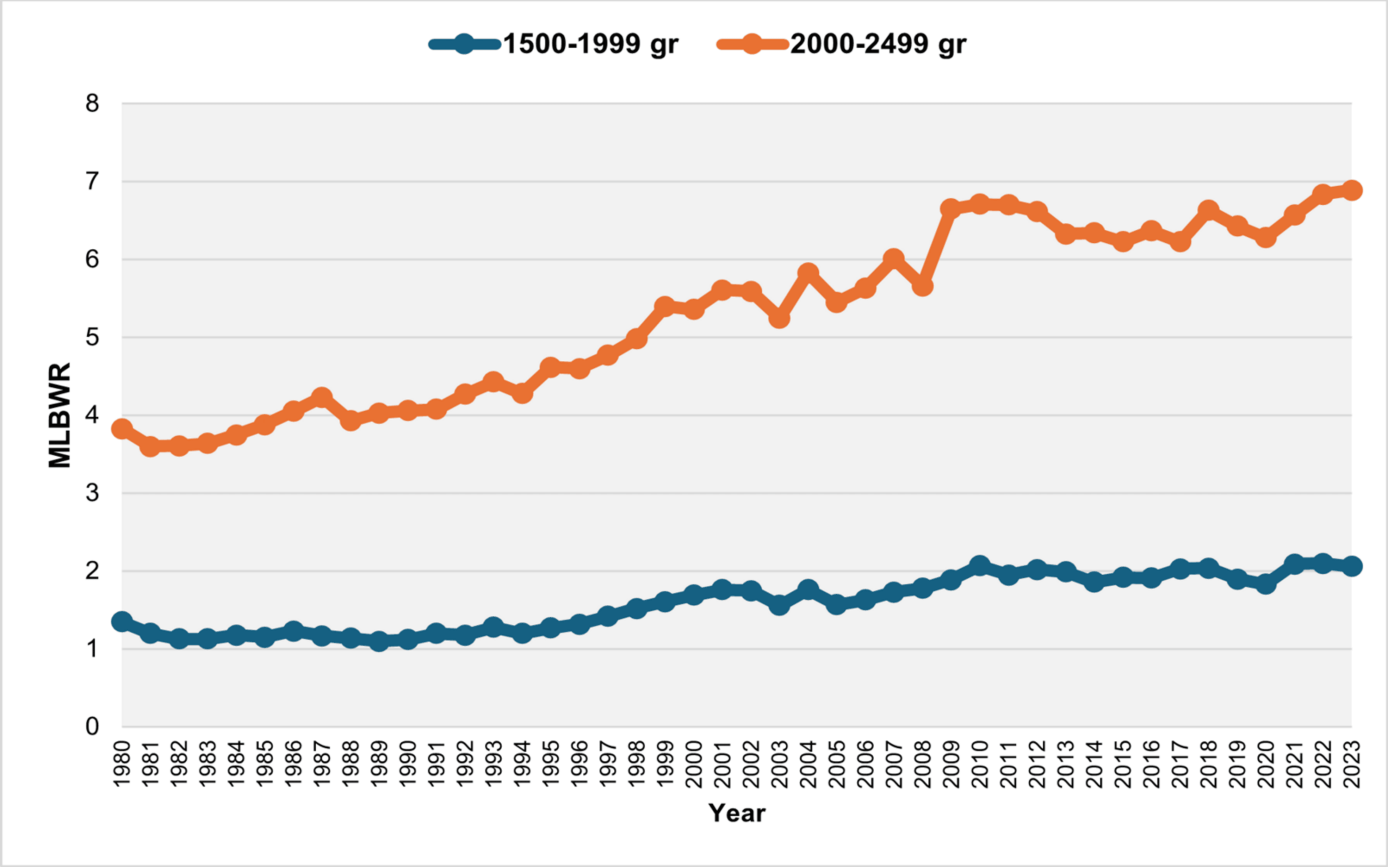
Moderately low birthweight rate (MLBWR) by category (1,500-1,999 g and 2,000-2,499 g) in Greece, 1980-2023

**Figure 5:**
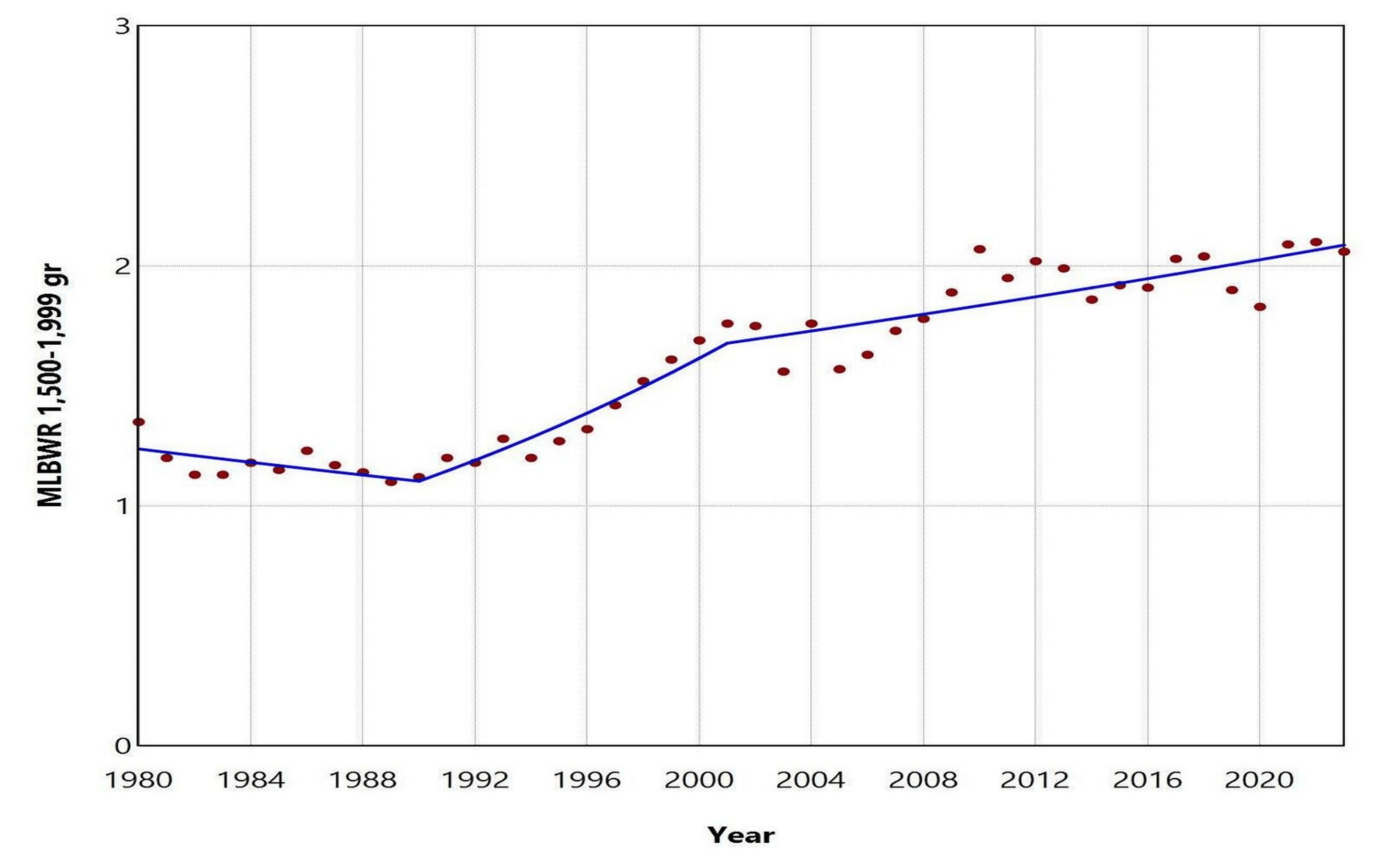
Trends in the 1,500-1,999 g category of the moderately low birthweight rate (MLBWR) in Greece, 1980-2023 Solid lines represent the estimated trends derived from joinpoint regression analysis.

**Figure 6:**
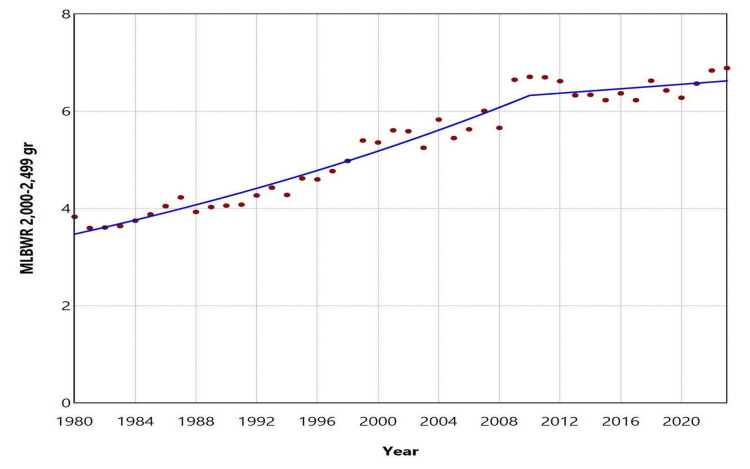
Trends in the 2,000-2,499 g category of the moderately low birthweight rate (MLBWR) in Greece, 1980-2023 Solid lines represent the estimated trends derived from joinpoint regression analysis.

Over the entire period from 1980 to 2023, the total number of moderately LBW neonates (1500 to 2499 grams) was 309,000, resulting in an overall MLBWR of 6.73%. The distribution of low birthweight newborns across the categories < 1500 g, 1500-1999 g, and 2000-2499 g was 12.5%, 20.2%, and 67.3%, respectively. The MLBWR for the 1500-1999 g category ranged from a low of 1.13% in 1982 and 1983 to a high of 2.10% in 2022. The trend showed a slight decline during 1980-1990, though it was marginally non-significant (APC = -1.1, 95% CI: -2.9 to 0.0, p = 0.054). This was followed by a rapid increase between 1990 and 2001, with an APC of 3.9 (95% CI: 2.9 to 7.9, p < 0.001), which continued at a slower pace from 2001 to 2023 (APC = 1.0, 95% CI: 0.5 to 1.3, p = 0.003). For the 2000-2499 g category, the MLBWR ranged from 3.60% in 1981 to 6.89% in 2023. The MLBWR in this category rose steadily over three decades (1980-2010: APC = 2.0, 95% CI: 1.8 to 2.3, p < 0.001) and remained essentially unchanged from 2010 to 2023 (APC = 0.4, 95% CI: -1.0 to 1.1, p = 0.417) (Figures 4-6, Tables 2, 3).

**Table 2:** Trends in the 1,500-1,999 g category of the moderately low birthweight rate in Greece, 1980-2023 Trends were calculated using joinpoint regression analysis.

Segment	Annual percent change	95% confidence interval	P-value
1980-1990	-1.1	-2.9 to 0.0	0.054
1990-2001	3.9	2.9 to 7.9	< 0.001
2001-2023	1.0	0.5 to 1.3	0.003

**Table 3:** Trends in the 2,000-2,499 g category of the moderately low birthweight rate in Greece, 1980-2023 Trends were calculated using joinpoint regression analysis.

Segment	Annual percent change	95% confidence interval	P-value
1980-2010	2.0	1.8 to 2.3	< 0.001
2010-2023	0.4	-1.0 to 1.1	0.417

## Discussion

This study examined official national data covering more than 4.5 million live births, providing a detailed overview of LBWR in Greece over a span of 44 years. The findings indicated a notable rise in LBWR during the 1990s and 2000s, with rates remaining persistently high in the years since. This trend represents a major concern for perinatal medicine and highlights an ongoing public health challenge in the country.

The time trend analysis, using the best-fitting model, identified three distinct phases in LBWR trends. During the 1980s, the LBWR remained relatively stable at low levels. However, from 1990 to 2010, the rate increased sharply, rising by an average of 2.3% annually. Between 2010 and 2023, the upward trajectory stabilized, but the rate persisted at high levels without any indication of decline. Since 2008, LBWR figures have consistently remained above 9%, and in the past two years (2022 and 2023), they exceeded 10%.

**Figure 7:**
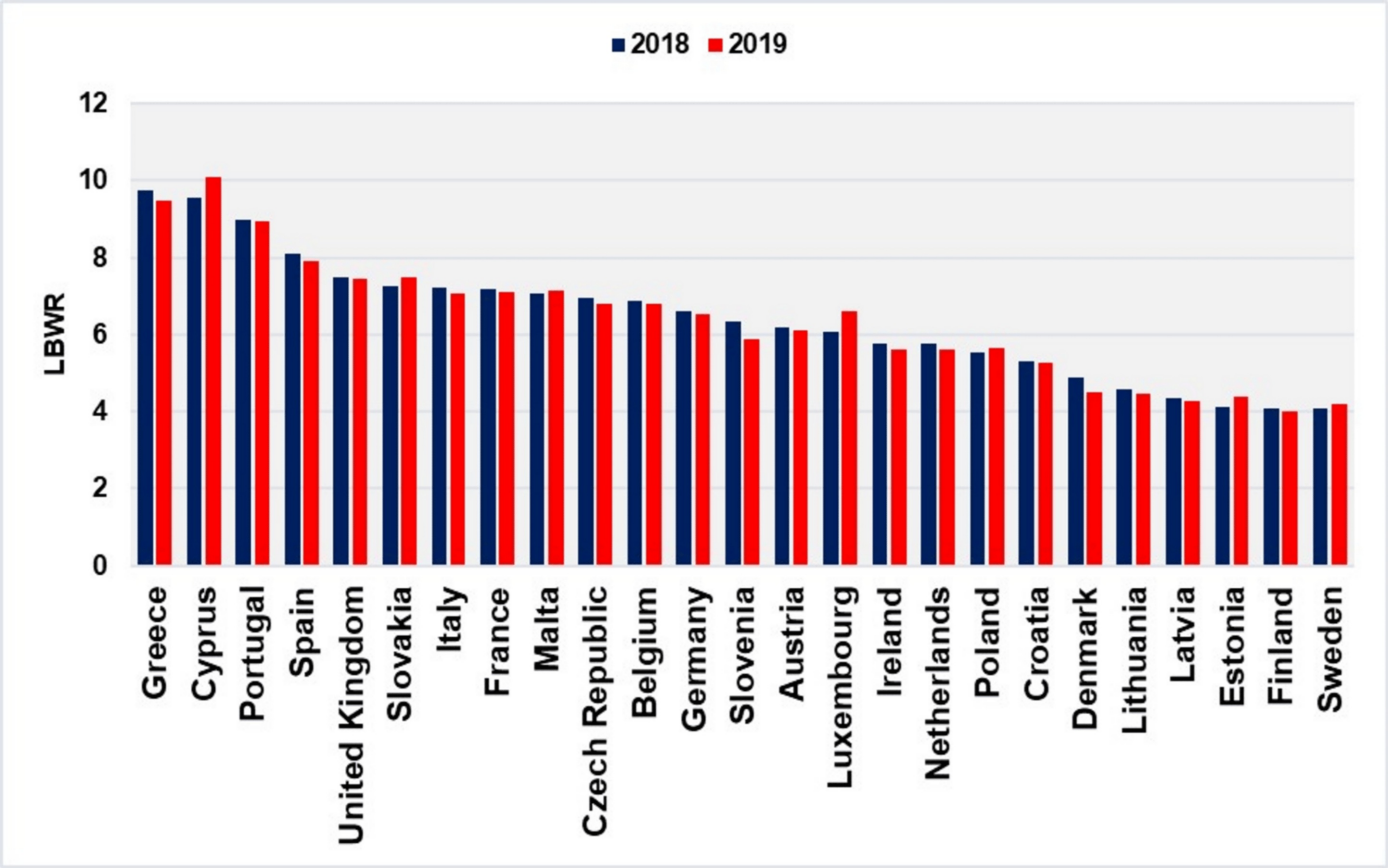
Low birthweight rates (LBWR) (per 100 live births) among 25 European Union countries, 2018 and 2019 Created by Nikolaos Vlachadis using data from EUROPERISTAT [9] for 24 countries, with additional data for Greece from this study. Data for Hungary, Bulgaria, and Romania are not included.

Greece ranks among the countries with the highest rates of LBW newborns in the developed world. Based on EUROPERISTAT data, Greece and Cyprus shared the highest LBWR rates within the European Union in 2018 and 2019. Specifically, Greece ranked first in 2018, followed by Cyprus in second place, while in 2019, it ranked second, with Cyprus taking the top position. The distribution among countries clearly shows that LBWR was highest in Mediterranean nations, moderate in Central and Western European countries, and lowest in Northern European countries. The median LBWR for European Union countries was 6.34% in 2018 and 5.87% in 2019, while Greece recorded significantly higher rates of 9.75% and 9.49%, respectively (Figure 7) [9].

**Figure 8:**
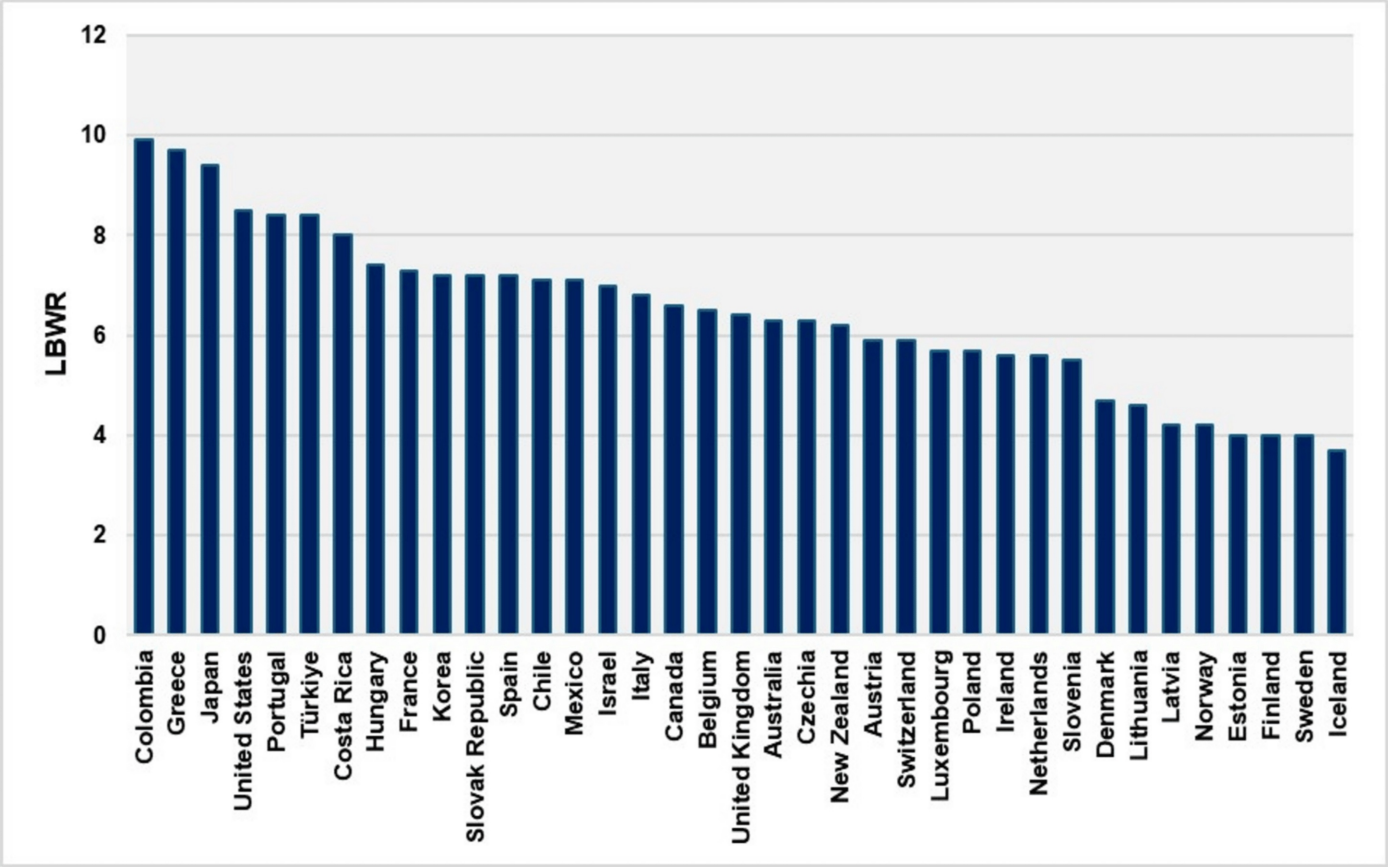
Low birthweight rate (LBWR) for the member countries of the Organisation for Economic Co-operation and Development (OECD), 2021 Created by Nikolaos Vlachadis using available data from OECD [10] for 37 countries, with additional data for Greece from this study. Data for Germany are not included.

Additionally, based on 2021 data, Greece had the second-highest LBWR among member countries of the Organisation for Economic Co-operation and Development (OECD). This distribution reveals that Greece, Colombia, and Japan were the only OECD countries with rates exceeding 9%, while the median LBWR for all OECD countries stood at 6.4% (Figure 8) [10].

Moreover, based on 2020 data, Greece had the second-highest proportion of LBW among 54 countries across Europe, North America, Australia, New Zealand, and Central Asia. In this comparative ranking, Turkey, Greece, and Bulgaria were the only countries with a double-digit LBWR. Furthermore, in 2022, the overall LBWR in the United States stood at 8.60%, while in Greece, it was 17% higher, climbing to 10.07% [11].

Subsequent analysis examined the trends in VLBWR and the two categories of MLBWR: 1,500-1,999 g and 2,000-2,499 g. The findings revealed that the VLBWR continued to increase at a steady rate of 0.9% annually over the four-decade study period. Additionally, since 2010, the MLBWR for newborns weighing 2,000-2,499 g has remained stable, while the subgroup of MLBW infants weighing 1,500-1,999 g maintained a statistically significant upward trend. In other words, the concerningly high yet stable trajectory of LBWR after 2010 has coincided with a persistent rise in the proportion of newborns weighing < 2,000 g, the group associated with the most severe complications.

The increasing trend in LBWR and its persistence at very high levels can primarily be attributed to unfavorable demographic factors related to fertility in the country. Greece has a high rate of births to mothers of advanced age, which is a significant risk factor for LBW [1,12]. Additionally, LBWR is strongly correlated with the incidence of preterm births. The parallel trajectories of LBW and preterm birth rates in Greece are evident, as both indicators showed a sharp upward trend during the 1990s and 2010s, with some attenuation after 2010 [7]. Given the close link to preterm birth rates, LBWR should also be viewed in the context of lax obstetrical practices regarding early induction of delivery without strict medical indications [7].

Both prematurity rates and LBWR are key complications of multiple pregnancies, which are at exceptionally high levels in Greece [7,13-15]. In 2022, in the United States, the LBWR among multiple births was more than eight times higher than that in singletons (57% vs. 7%, respectively) [11]. The impact of preterm births and multiple births on the trends and values of LBWR in Greece warrants further evaluation. Additionally, a strong positive correlation has been observed between LBWR and worsening socioeconomic conditions [5,6]. In Greece, the onset of the severe economic recession in 2008 was accompanied by an immediate and sharp deterioration in LBWR, which increased significantly by 19% from 2008 to 2010, while the VLBWR increased by 23%, reaching an all-time high in 2010. The upward trend in LBWR during the country's economic crisis has been extensively documented in the literature [16-19]. Interestingly, sociodemographic factors have shown only a small effect on LBWR values in Greece [20,21].

In the present study, to our knowledge, the rates of LBW newborns in Greece were presented for the first time in the literature, highlighting their significant increase over recent decades. Furthermore, a comparison with international data revealed that Greece maintains the highest LBWR among high-income countries. Of particular concern is the finding that, while the upward trend in LBWR has recently flattened, the VLBWR and the MLBWR 1,500-1,999 g continued to rise. These findings underscore LBW births, in conjunction with the country’s already high preterm birth rates, as a significant public health threat in Greece, creating a large cohort of vulnerable neonates every year [22].

LBW births are the primary risk factor for neonatal and infant mortality [23]. Additionally, they represent a major risk factor for impaired development and metabolic disorders in adulthood, directly linked to cardiovascular complications [4]. The study of long-term trends shows that Greece is far from achieving the WHO targets for reducing LBWR. Interventions are urgently needed, focusing primarily on reducing preterm births, as evidence-based preventive strategies for fetal growth restriction remain challenging to implement. Efforts should target the application of evidence-based medical practices to reduce multiple pregnancies resulting from assisted reproduction, prevent pregnancy complications such as preeclampsia, and ensure adequate antenatal care for all pregnant women. Public health interventions should also prioritize the prevention of maternal morbidity, with a particular focus on socially and economically disadvantaged populations [7,22,24].

The present study is strengthened by the analysis of a very large dataset comprising over 4.5 million live births spanning more than four decades, along with the validity of birthweight data derived from official birth certificates. However, this study is limited by the nature of LBWR, which, although well-established for studying birthweight and identifying neonates at risk in large populations [1,4], relies on a standard cutoff of 2,500 g without accounting for gestational age [2,3]. Additionally, the present study was limited to the analysis of birthweight data and lacked individual-level variables, such as maternal age, birth plurality, gestational duration, or socioeconomic factors (e.g., maternal education). Further research should aim to elucidate the role of these factors in shaping LBWR in Greece, with particular emphasis on two key determinants: multiple pregnancies and preterm births. In addition, a deeper exploration into maternal health care policies and lifestyle factors could provide valuable insights into the unfavorable LBWR trends observed in the country. Finally, monitoring the long-term trends of LBWR must continue diligently, alongside further research into the causes of preterm births and LBW, especially in the context of implementing evidence-based obstetrical practices.

## Conclusions

The present study provides a comprehensive time trend analysis of LBWR in Greece over a 44-year period, from 1980 to 2023. The findings highlight a sharp increase in LBWR during the 1990s and 2000s, with rates stabilizing at record-high levels in recent years, surpassing 10% in 2022 and 2023. Greece consistently ranks among the highest in LBWR within developed countries, underscoring a significant public health challenge. The rise in LBWR is likely associated with unfavorable maternal demographic trends, high rates of multiple pregnancies and preterm births, and socioeconomic disparities exacerbated by the recent financial crisis. Despite the recent flattening of overall LBWR, the persistent rise in the VLBWR and the MLBWR for neonates weighing 1,500-1,999 g is particularly concerning, as these infants face the highest risks. These findings are critical for public health stakeholders in the country, as targeted interventions are urgently needed to reduce the burden of low birthweight and the associated neonatal and long-term health complications.
